# Immunologic Profiling of the Atlantic Salmon Gill by Single Nuclei Transcriptomics

**DOI:** 10.3389/fimmu.2021.669889

**Published:** 2021-05-04

**Authors:** Alexander C. West, Yasutaka Mizoro, Shona H. Wood, Louise M. Ince, Marianne Iversen, Even H. Jørgensen, Torfinn Nome, Simen Rød Sandve, Samuel A. M. Martin, Andrew S. I. Loudon, David G. Hazlerigg

**Affiliations:** ^1^Arctic seasonal timekeeping initiative (ASTI), Department of Arctic and Marine Biology, UiT – The Arctic University of Norway, Tromsø, Norway; ^2^Unit of Animal Genomics, GIGA Institute, University of Liège, Liège, Belgium; ^3^Department of Pathology and Immunology, Faculty of Medicine, University of Geneva, Geneva, Switzerland; ^4^Centre for Integrative Genetics (CIGENE), Department of Animal and Aquacultural Sciences (IHA), Faculty of Life Sciences (BIOVIT), Norwegian University of Life Sciences (NMBU), Ås, Norway; ^5^Institute of Biological and Environmental Sciences, University of Aberdeen, Aberdeen, United Kingdom; ^6^Division of Diabetes, Endocrinology & Gastroenterology, School of Medical Sciences, Faculty of Biology, Medicine and Health, University of Manchester, Manchester, United Kingdom

**Keywords:** Atlantic salmon *(Salmo salar)*, smoltification, photoperiod, immune cells, gill, single nuclei RNA sequencing

## Abstract

Anadromous salmonids begin life adapted to the freshwater environments of their natal streams before a developmental transition, known as smoltification, transforms them into marine-adapted fish. In the wild, smoltification is a photoperiod-regulated process, involving radical remodeling of gill function to cope with the profound osmotic and immunological challenges of seawater (SW) migration. While prior work has highlighted the role of specialized “mitochondrion-rich” cells (MRCs) and accessory cells (ACs) in delivering this phenotype, recent RNA profiling experiments suggest that remodeling is far more extensive than previously appreciated. Here, we use single-nuclei RNAseq to characterize the extent of cytological changes in the gill of Atlantic salmon during smoltification and SW transfer. We identify 20 distinct cell clusters, including known, but also novel gill cell types. These data allow us to isolate cluster-specific, smoltification-associated changes in gene expression and to describe how the cellular make-up of the gill changes through smoltification. As expected, we noted an increase in the proportion of seawater mitochondrion-rich cells, however, we also identify previously unknown reduction of several immune-related cell types. Overall, our results provide fresh detail of the cellular complexity in the gill and suggest that smoltification triggers unexpected immune reprogramming.

## Introduction

During its life cycle, the Atlantic salmon migrates between fresh and seawater environments (1). Atlantic salmon eggs hatch in freshwater streams where they develop for 1-4 years. On reaching a critical size threshold, immature fish known as ‘parr’ are sensitized by several weeks of winter photoperiod (day-lengths), after which, exposure to increasing photoperiods stimulates the parr to transform into a ‘smolt’ ready for migration to sea (2). This process, known as smoltification, is mediated by endocrine factors that collectively deliver extensive phenotypic remodeling, leading to overt changes in length, weight, silvering, migratory behavior, immune function and osmoregulatory capacity, dependent on gill physiology (1).

The salmonid gill is a complex multifunctional organ, essential for gas exchange, nitrogenous waste excretion, pH balance and osmoregulation (3). It is also a major mucosal immune barrier harboring a dedicated lymphoid tissue termed gill associated lymphoid tissue (GIALT) (4, 5) which is rich in T cells, natural killer cells and macrophages. Although it is known that smoltification suppresses immune function, little, if anything, is known about which immune cell types in the gill are modified (6). Structurally, the gills are arranged in symmetrical arches, each of which are populated by numerous filament structures, which are themselves densely flanked with lamellae. The gill is composed of seven major cell types (7). Pavement cells (PVCs) have an enlarged surface area on the apical membrane, and form the majority of the epithelium (8). Pillar cells (PCs), which are structural cells, define the blood spaces within the lamellae (9). Goblet cells (GCs) reside in the filament epithelium and excrete mucus (10). Non-differentiated progenitor cells (NDCs) colonize basal and intermediate layers of the gill epithelium (11). Chemosensory neuroepithelial cells (NECs) lie along the length of the efferent edge of the gills and are innervated by the central nervous system (12). Mitochondrion-rich cells (MRCs) and their adjacent accessory cells (ACs) are located at the trough between two lamellae where they abundantly express the channels and pumps required to maintain the osmotic gradients between blood plasma and both fresh- and seawater (13–15).

Smoltification induced increases in cortisol and growth hormone, as well as declines in prolactin drive conversion of the Atlantic salmon gill from a freshwater-adapted organ to a seawater-adapted organ. This change in endocrinology coincides with a switch in anatomical and molecular phenotypes of MRCs and ACs, and these have formed the major focus of smoltification of gill physiology (1, 14, 16). In the gills of fish living in freshwater, Na^+^ ions are taken up by proton exchange across the apical membrane of MRCs and then transported into the blood *via* the sodium potassium ATPase (NKA) on the basolateral membrane (17–19). Cl^-^ ions, meanwhile, are exchanged or channeled across the apical membrane then enter the blood through an undefined channel (20–23). In saltwater adapted gills, NKA in the basolateral membranes of MRCs generates a chemical and electrical gradient, motivating both loss of Cl^-^ ions *via* the smoltification-induced apical CFTR channels and paracellular escape of Na^+^ ions (15, 24) [reviewed in (25)].

While changes in MRC function are undoubtedly of central importance for the ion regulatory changes which take place during smoltification, the remodeling of gill phenotype likely extends far beyond these facets (26, 27). During this time the fish are exposed to novel pathogens to which they have not previously been exposed and it is hypothesized that reorganization of the gill immune system needs to coincide with the required physiological changes (6, 28, 29). Here we resolved the complexities of smoltification-driven changes in gill cytology using a single-nuclei RNAseq strategy, exploring the transcriptional responses to smoltification and seawater transfer at individual nuclei-level resolution, with a particular emphasis on immune cell abundance and transcriptional expression.

## Material and Methods

### Animal Welfare Statement

The Atlantic salmon smoltification experiment was conducted as part of the routine, smolt production at Kårvik havbruksstasjonen, and was approved by the Norwegian Animal Research Authority (NARA) for the maintenance of stock animals for experiments on salmonids. This is in accordance with Norwegian and European legislation on animal research.

### Experimental Design

Atlantic salmon (*Salmo salar*, Aquagene commercial stain) were raised from hatching in freshwater, under continuous light (LL, > 200 lux at water surface) at ambient temperature (~10°C). Juvenile salmon were housed in 500 L circular tanks and fed continuously with pelleted salmon feed (Skretting, Stavanger, Norway). At seven months of age parr (mean weight 49.5g) were sampled for T1 (experiment start). Two days later remaining parr were equally distributed between two 100L circular tanks, and over the next seven days the photoperiod was incrementally reduced to a short photoperiod (SP, 8h light:16h darkness). T2 sampling occurred on experimental day 53 (44 days on SP), remaining parr were transferred back to LL on experimental day 60. T3 sampling occurred on experimental day 110 (50 days after return to LL), then a sub-cohort of fish were netted out and transferred to full strength seawater for 24h before the final T4 collection.

### RNAseq Analysis

Gill samples were collected and RNA extracted as described in Iversen et al. (27). Sequencing libraries were prepared using the TruSeq Stranded mRNA HS kit (Illumina). Library mean length was determined by a 2100 Bioanalyzer using the DNA 1000 Kit (Agilent Technologies) and library concentration was measured with the Qubit BR Kit (Themo Scientific). Each sample was barcoded using Illumina unique indexes. Single-end 100bp sequencing of sample libraries was carried out on an Illumina HiSeq 2500 at the Norwegian Sequencing Center (University of Oslo, Oslo, Norway). Cutadapt (30) was used to remove sequencing adapters, trim low quality bases, and remove short sequencing reads using the parameters -q 20 -O 8—minimum-length 40 (version 1.8.1). Quality control of the reads were performed with FastQC software. Mapping of reads to reference genome was done using STAR software (ver. 2.4.2a) (31). HTSEQ-count software (version 0.6.1p1) was used to generate read count for annotated genes (32). Raw counts were analyzed using EdgeR (ver. 3.30.0), using R (ver. 4.0.2) and RStudio (ver. 1.1.456). A quasi-likelihood F-test with exhaustive intergroup contrasts was used to identify differential expressed genes between T1-T3 samples, an FDR threshold was set to <0.01. Clustering analysis was performed using Pearson correlation, and heatmaps rendered using the R package pheatmap. An exact test was performed to identify differential expressed genes between T3 and T4. RNAseq data is available from the European nucleotide archive (PRJEB34224).

### Single Nuclei RNAseq Analysis

Our choice of single nuclei RNAseq (snRNAseq) rather than single cell RNAseq (scRNAseq) allowed us to use frozen samples. The use of frozen samples permits consistent dissociation of fibrous gill tissue, prevents gene expression changes provoked by the dissociation of living cells, and allows for parallel library preparation of our longitudinal study samples (33). Comparison between snRNAseq and scRNAseq report broadly comparable gene detection but it should be noted that nuclear depleted genes are less visible to a snRNAseq analysis (34, 35).

Gills for single nuclei analysis were snap frozen on dry ice and stored at -80°C. Duplicate samples were processed for T1-T4. Nuclei were released by detergent mechanical lysis, then samples were homogenized (30s) and nuclei isolated by sucrose gradient (36). Libraries were created using Chromium Single Cell 3′ GEM, Library & Gel Bead Kit v3 (10x technologies) using a NextSeq500 by University of Manchester genomic technology core facility (UK). Raw data was processed using Cell Ranger (10x Technologies, ver. 3.1.0), where the count command generated counts per cell. The cell count was 2355.5 ± 539.8 (SD) for each sample and the pooled duplicate cell count for T1-T4 was 4771 ± 163.6 (SD). The NCBI ICSASG_v2 genome was used for alignment, with gene annotations from the NCBI Salmo salar Annotation Release 100. The R package Seurat (ver. 3.1.5) was used to perform an integrated analysis using all snRNAseq data (37), further details in results and discussion. Raw and processed data is available from GEO data archive (GSE166686).

### Gene Ontology Analysis

Human orthologs to Atlantic salmon genes were identified by generating protein sequence homology based orthogroups using the Orthofinder pipeline (38). Where possible, this links Atlantic salmon genes to their human gene counterpart through the shortest distance in ortholog gene trees. Human genome nomenclature consortium (HGNC) identifiers were then used to infer gene ontology to Atlantic salmon genes cohorts using the Consensus Path Database over-representation analysis adjusted to a background list of all genes expressed in the analysis (39). Taken together these data form a useful indication of the concerted function of the gene lists, however, we encourage a degree of skepticism in the roles of individual genes, few of which have been tested for isofunctionality with their orthogroups (40).

## Results

### A Single-Nuclei Survey of Atlantic Salmon Gill Cells

**Figure 1:**
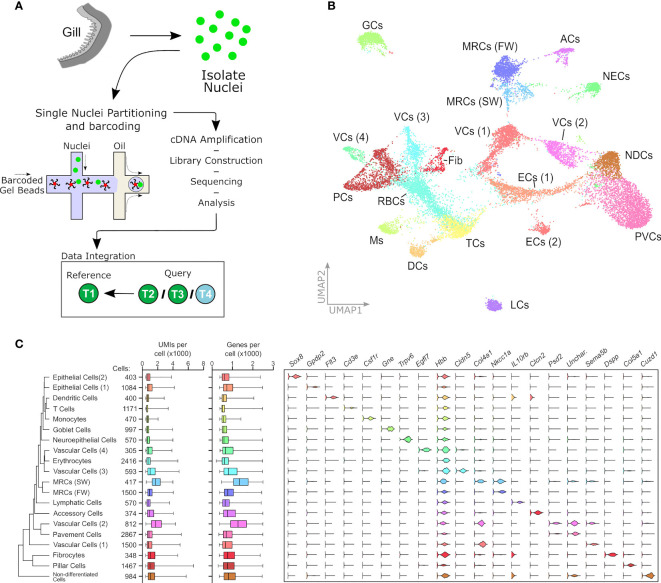
Single nuclei RNAseq analysis of Atlantic salmon gill tissue. **(A)** Gill tissue processing. Pooled duplicates from all T2, T3 and T4 collection points are integrated against T1 as a reference set. **(B)** UMAP plot of pooled cell data from 18844 cells representing eight samples from four collection states. The plot indicates 20 separate cell clusters. **(C)** Expression of marker genes in 20 cell clusters. From left to right: hierarchical relatedness of difference cell clusters; total cells in each cluster; UMI number in each cell cluster; gene features in each cell cluster; violin plots showing expression pattern of marker genes for each cluster. ACs, accessory cells; DCs, dendritic cells; ECs, epithelial cells; fib, fibrocytes; GCs, goblet cells; LCs, lymphatic cells; Ms, monocytes; MRC, mitochondrion-rich cells; NDCs, non-differentiated cells; PVCs, pavement cells; RBCs, red blood cells (erythrocytes); TCs, T cells; VCs, vascular cells.

We profiled 18,844 individual nuclei from eight Atlantic salmon gill samples from four smolt developmental states (**Figure 1A**). To define the nuclei cluster structure across developmental states we pooled duplicate samples and integrated data between all four states. We next identified anchors: cells that represent shared biological states across datasets. Anchors were then used to calculate “correction” vectors allowing all fours states to be jointly analyzed as an integrated reference (37). Unsupervised graph clustering partitioned the nuclei into 20 clusters, which are defined by the correlative co-expression of a list of marker genes (**Supplementary Table 1**). We then visualized these data using a uniform manifold approximation and projection (UMAP) dimension reduction technique (**Figure 1B**). To assign a cell identity to each cluster we identified expression of cell-specific marker genes where possible, then complemented this approach using an unbiased gene ontology analysis (**Figures 1B, C**).

**Figure 2:**
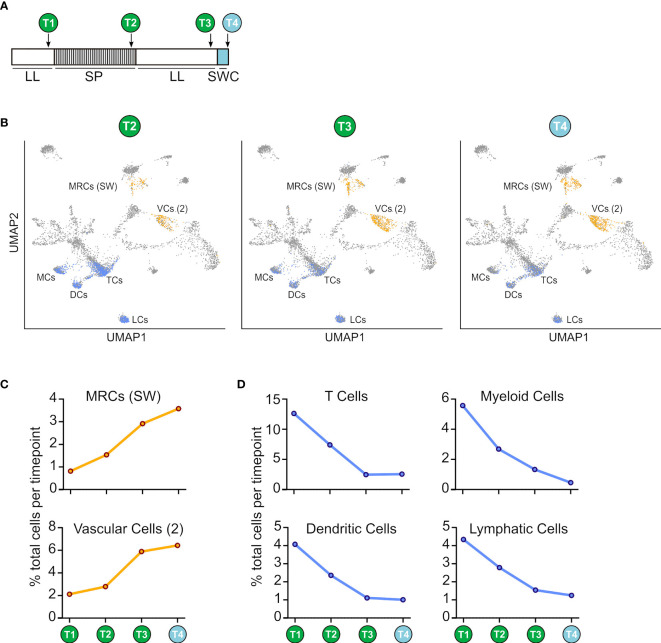
Comparative abundance of cell clusters at different sampling points. **(A)** Experimental design. Fish were kept in constant light (LL) from hatching then transferred to short photoperiod (SP; 8L:16D) for 8 weeks before being returned to constant light (LL) for 8 weeks. Finally the fish were transferred to sea water for 24h. Sample points are indicated T1-T4. **(B)** Subset of cell clusters from T2, T3 or T4 (orange and blue dots) overlaid on T1 cells (grey dots). **(C)** Increasing abundance of sea-water mitochondrion-rich cells (MRCs SW) and vascular cells (VC 3) during smoltification **(D)** Decreasing abundance of leukocytes and immune-associated cells during smoltification.

**Figure 3:**
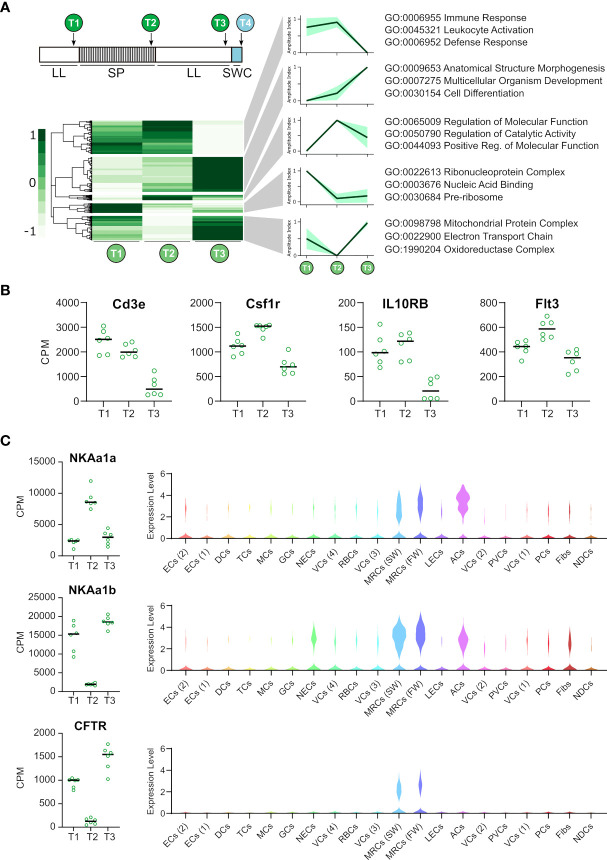
Photoperiodic changes in gill gene expression and localized cell cluster expression. **(A)** Heat map representing 9746 genes differentially regulated (FDR <0.01) from T1-T3. Regulatory patterns for 5 major cluster are shown as amplitude index and 95% confidence limits. Major gene ontology terms for each cluster are shown. **(B)** RNAseq data for immune-associated genes differentially expressed by smoltification (FDR <0.01). **(C)** RNAseq data for “classical” smoltification-related genes and violin plots showing their cluster specific expression.

The most well-described gill cells are the MRCs and ACs, which are clearly separated from other gill cell types by their abundant expression of the osmotic regulators *NKAa1a* and *NKAa1b* sodium-potassium ATPase subunits (for details see **Figure 3C**). The MRCs were highlighted within this subset by their shared expression of the sodium-potassium-chloride co-transporter *Nkcc1a*. We further discriminated the SW population of MRCs from the FW cluster by their increasing abundance through smoltification (see **Figure 2**), and through the specific expression of a collagen alpha-chain gene, *Col4a1*, which is shared in expression with two vascular cell (VC1 and VC2) groups, highlighting the developmental heritage of the SW MRC cluster (**Figure 1C**) (3). Interestingly, the AC cluster was characterized by its expression of *Slc26a6*, an apical membrane Cl^-^/HCO_3_ exchanger, heretofore misassigned to MRCs (**Figure 1C**) (41, 42).

Goblet cells were identified by the specific expression of the mucin gene *Muc5ac* (**Figure 1C**) (43). Cluster identity was supported by the association of enriched GO terms for ‘vesicle’ and ‘secretion by cell’ (**Supplementary Table 1**).

Erythrocytes were identified due to their expression of diverse hemoglobin subunits including *Hbb* (**Figure 1C**). We were interested to note that the markers defining the erythrocyte population, including beta-globin, were expressed widely among all cell types. It is unclear what role extra-erythroid hemoglobin plays in the gill, however, mammalian studies suggest that hemoglobin, in addition to its oxygen carrying capacity, may play an antimicrobial role (44). As a major mucosal immune barrier, this capacity may be pertinent to the gill (5).

Of great interest we also highlighted several immune cell clusters. The T cell cluster was identified by the classical marker *Cd3e* (**Figure 1C**) (45), and was enriched for the GO term ‘thymic T cell selection’ (**Supplementary Table 1**). A dendritic cell-like cluster was identified by *Flt3* (46), *Xcr1* (47) and *CD209* (48) expression, although of note, this cluster also expressed *Itgae* which is more typically associated with T cells (**Figure 1C** and **Supplementary Table 1**) (49). We further identified a monocyte-like cluster based on the expression of the monocyte markers *ACP2* and *C1QA* (50), although this cluster also likely subsumes neutrophil cells due to the presence of the *CSF3R* (51) and *LAMP2* (52) marker genes (**Supplementary Table 1**). Lastly we identify a lymphatic cell population defined by typical *Prox1* expression (**Figure 1C**) (53).

We defined a neuroepithelial cell cluster by its expression of *Notch1* (54), *Occludin* (55) and *Hes1* (56) markers in union with the enriched GO terms for ‘cellular response to stimulus’ and ‘signal transduction’ (**Supplementary Table 1**). The remaining eight cell types were broadly defined as clear molecular indicators were not found. A cluster of ‘non-differentiated cells’ was tentatively defined by the GO enrichment for ‘desmosome’ a structure typical to this cell type (**Supplementary Table 1**) (7). Two epithelial cell clusters were identified due to their high degree of relatedness, expression of tight junction and cell adhesion molecules including Cld4 (57) and PCDH11X (58), and enrichment of GO terms including ‘keratinization’. Four vascular cell clusters were described by their common GO enrichment for ‘tube development’ and ‘blood vessel development’ (**Supplementary Table 1**). We indicate a potential fibrocyte population due to its otospirilin expression (**Supplementary Table 1**) (59), and a pillar cell cluster characterized by its diverse collagen expression and enriched GO terms for ‘extracellular matrix’ (**Supplementary Table 1**). Lastly we define a pavement cell cluster by the high abundance of the cluster and its GO enrichment for the terms ‘apical junction complex’ and ‘basolateral plasma membrane’ (**Supplementary Table 1**). The novel populations of fibrocyte-like cells, and several types of vascular- and endothelial-like cells that partitioned across several clusters, together suggesting greater complexity in gill cytology that previously appreciated (**Figures 1B, C**).

### Major Changes in Cell Composition During Smoltification

To understand how gene expression and cellular complexity changes within the gill during smoltification and seawater transfer we compared the snRNAseq profiles at different developmental points [**Figure 2A** and **Supplementary Table 2**; for confirmation of smolt status see (27)]. The abundance of six nuclei clusters changed dramatically (>3 fold change in percentage abundance) during smoltification (**Figure 2B**). SW MRCs increased in proportion steadily from T1-T4, consistent with previous descriptions of Atlantic salmon gill physiology (60). We also observed a marked increase in vascular cell (2) number, with the major differences occurring between T2 and T3, suggesting that this vascular cell cluster proliferates in line with growth rates (**Figure 2C**). Interestingly, four immune-related nuclei clusters representing T cells, monocyte cells, dendritic cells and lymphatic cells fell dramatically during smoltification (**Figure 2D**). Changes in cell abundance occurred with a similar profile in all immune-associated cell clusters, with consistent decline observed between T1-T3. In contrast, 24h SW transfer does not appear to affect immune-cell abundance directly (**Figure 2D**). These results highlight the complex and dynamic changes in cellular composition that occur in the gill during smoltification.

### Nuclei Cluster-Specific Expression of Smoltification-Associated Factors

Next, we wanted to identify cluster types where smoltification is associated with cluster-specific gene regulation. As our snRNAseq dataset represented duplicate samples at each developmental point, we cross referenced our snRNAseq analysis with whole gill RNAseq analysis of T1-T3 (n = 6), identifying 9746 genes differentially regulated by smoltification (quasi-likelihood F-test with exhaustive intergroup contrasts, FDR <0.01). Pearson correlation clustering of these genes resolved five major clusters that were associated with immune response, structural morphogenesis, catalytic activity, ribonucleoprotein complexes and mitochondrial respiration (**Figure 3A**). Given the conspicuous decrease in immune cell abundance seen in our snRNAseq experiment, we data-mined our RNAseq dataset to track the expression of the marker genes for the T cell (Cd3e), monocyte (Csf2r), LEC (Il10rb) and DC (Flt3) clusters (**Figure 3B**). In accordance with our snRNAseq analysis each of these factors are reduced after smoltification, supporting the evidence that immune cells are depleted during this process. A number of “classical” smoltification-related genes was also identified and localized to specific cell types (**Figure 3C**). As expected, CFTR was highest under constant light (LL), and was highly localized in expression to MRCs. We also identified the reciprocal regulation of sodium-potassium ATPase subunits, specifically, suppression of NKAa1a and increase in NKAa1b (14). Inspection of cellular localization within our snRNAseq dataset showed that expression of these genes were, as anticipated, highest within the MRCs and ACs (**Figure 3C**).

Our previous work identified genes whose expression are predicated on exposure to several weeks of short-photoperiod exposure (27). In Atlantic salmon, these “winter-dependent” genes are analogous to vernalization dependent genes in *Arabidopsis (*61), where a dosage of exposure to a winter-like stimulus (in *Arabidopsis*, cold; in Atlantic salmon, short photoperiod) controls the presentation of a seasonal phenotype under summer-like stimulus (in *Arabidopsis*, warmth and long days; in Atlantic salmon, long photoperiod). Winter-dependent genes are therefore intrinsically linked to unidirectional smolt development, and may play a mechanistic role in pre-adaptation of the gill for seawater migration. Surprisingly, canonical markers of smolt status, including the reciprocal expression of NKA subunits, are not winter-dependent. Rather than indicating life history progression, NKA subunit expression correlates directly to photoperiod, meaning that their usefulness in asserting smolt status is flawed (27).

Using our RNAseq dataset we identified novel, winter-dependent genes which we then isolated from our snRNAseq dataset to identify the cell clusters that express these factors (**Supplementary Figure 1**). Of particular interest was Cuzd1, a gene associated with tumorigenesis, as well as prolactin-induced JAK/STAT5 signaling during mammary gland development in mice, and muscle growth in zebrafish (62–65). The induction of Cuzd1 within non-differentiated cells of the Atlantic salmon gill suggests Cuzd1 is important in gill development during smoltification, and may hint at a role for prolactin signaling. We also identified Rhag, a transporter associated with erythrocytes in mammals, but expressed in the teleost gill where it is thought to regulate ammonium excretion (66–68). Its predominant expression within the vascular cell (VC 3) cluster suggests this cluster plays a specialized role in ammonium balance within the Atlantic salmon gill (69). We also highlight Hg2a (CD74), a multifunctional protein best characterized as a chaperone during mammalian MHCII antigen presentation but also important for endosomal trafficking, cell migration and cellular signaling (70, 71). Hg2a is expressed in the gills of other teleosts however little is known of its function within this context (72, 73). The striking abundance of Hg2a transcripts in our analysis and its common expression in all cell clusters suggests it plays a valuable role in salmonid gill function. Taken together our data show that the phenotypic change driven by smoltification is diverse and engages all gill cells.

### Cell Cluster-Specific Expression of Seawater Transfer-Associated Factors

**Figure 4:**
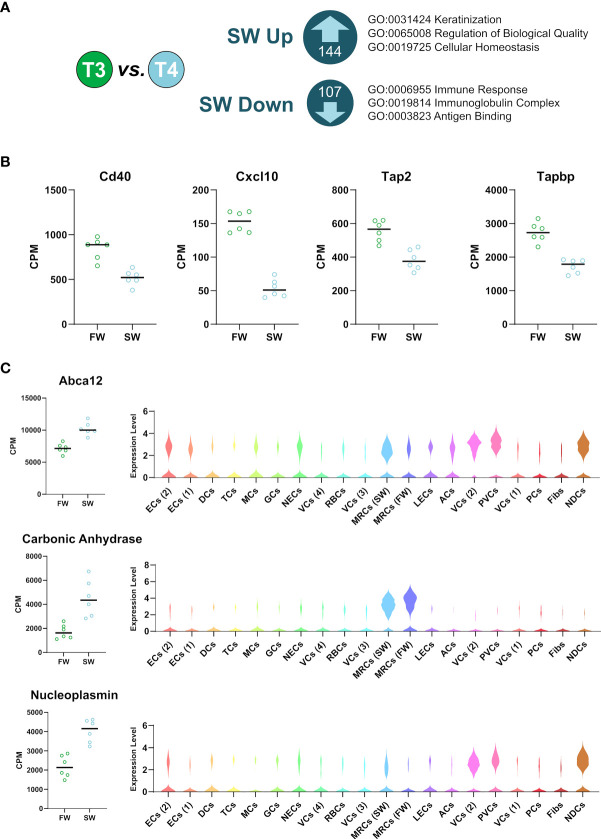
Sea-water transfer-associated changes in gill gene expression and localized cell cluster expression. **(A)** Genes differentially regulated (FDR <0.01) by 24h seawater transfer. Major gene ontology terms for each cluster are shown. **(B)** RNAseq data for immune-associated genes suppressed by seawater transfer (FDR <0.01) **(C)** RNAseq data for sea-water transfer-related genes and violin plots from the snRNAseq dataset showing their cluster specific expression.

Smoltification manifests when the Atlantic salmon smolts migrate downstream and arrive in the marine environment, thereby committing to an oceanic phase of the life cycle (1). To gain insight into this critical step in smolt gill remodeling we identified 144 induced and 107 suppressed genes (whole gill RNAseq, FDRE<0.01; **Supplementary Table 3**) after exposure to seawater for 24h. Gene ontologies showed the induced gene cohort was significantly associated with keratinization (**Figure 4A**) and the suppressed gene cohort was related to immune function, including the key immune regulators CD40, CXCL10, TAP and TAPBP (**Figure 4B**).

We then cross-referenced the seawater induced genes (**Figure 4A**) with our snRNAseq data to identify cluster-specific gene regulatory responses (**Figure 4C**). For example, we localize the expression of an enzyme involved in both ionic and acid/base balance, carbonic anhydrase, to MRCs (26, 74). We also show that ATP-binding cassette sub-family A member 12 (Abca12), a gene important in epidermal lipid barrier formation (75), is broadly expressed, but particularly concentrated in MRCs (SW), pavement, vascular, and non-differentiated cells. Interestingly, we show that a protein chaperone that helps regulate chromatin state, nucleoplasmin (76, 77), is expressed specifically in non-differentiated, vascular and pavement cells groups, suggesting that these cell types undergo a change in chromatin status under seawater exposure.

## Discussion

Our results bring insightful cellular resolution to the complexity of the Atlantic salmon gill and the compositional changes that occur during smoltification. Of particular interest was the suppression of immune cell types, which correlates with reduction in immune-related genes and suppression of immune function during smoltification and seawater transfer (6, 29, 78). These data are a puzzle. The marine environment is awash with parasites, bacteria and viruses to which the salmon is potentially vulnerable, so loss of immune function would make little sense. Future work should focus on why and how the immune system is affected in aquaculture, and should include analyses of other important immune tissues to contextualize the response to smoltification beyond that which we report in the gill. Indeed, other studies suggest a systemic suppression of the immune system during smoltification and seawater transfer, including in the head kidney and intestine (38). Conceivably these data point towards an adaptive immunological reprogramming that helps to avoid immune shock when the salmon transition between the distinctive pathogen complements of fresh- and seawater habitats (79, 80). Alternatively, artificial smolt production may drive abnormal immunosuppression. The constant light routinely used to stimulate smolts would profoundly undermine the immune defenses of mammals *via* disruption of the circadian clock (81).

Our data also show that smoltification-driven transcriptional regulation occurs not only in MRCs and ACs, but also in other distinctive cell types including pavement cells, vascular cells and non-differentiated cells. We anticipate that novel gene function within the context of cell function will be a priority for future investigation, and will be assisted by the novel suite of marker genes which we present here.

## Data Availability Statement

The datasets presented in this study can be found in online repositories. The names of the repository/repositories and accession number(s) can be found in the article/**Supplementary Material**.

## Ethics Statement

Ethical review and approval was not required for the animal study because the Atlantic salmon smoltification experiment was conducted as part of the routine, smolt production at Kårvik havbruksstasjonen. This is approved by the Norwegian Animal Research Authority (NARA) for the maintenance of stock animals for experiments on salmonids in accordance with Norwegian and European legislation on animal research.

## Author Contributions

Conceptualization, AW, YM, EJ, AL, and DH. Resources, AW, YM, and MI. Investigation, AW, YM, MI, EJ, and DH. Formal Analysis, AW, YM, LI, SM, TN, and SS. Visualization, AW, and SW. Writing – Original Draft, AW, and SW. Writing – Review and Editing, All. Supervision, AL and DH. Project Administration, AL and DH. Funding Acquisition, AL and DH. All authors contributed to the article and approved the submitted version.

## Funding

AW is supported by the Tromsø forskningsstiftelse (TFS) grant awarded to DH (TFS2016DH). The Sentinel North Transdisciplinary Research Program Université Laval and UiT awarded to DH supports this work. SW is supported a grant from the Tromsø forskningsstiftelse (TFS) starter grant TFS2016SW. Experimental costs were covered by HFSP grant “Evolution of seasonal timers” RGP0030/2015 awarded to AL and DH. Storage resources were provided by the Norwegian National Infrastructure for Research Data (NIRD, project NS9055K).

## Conflict of Interest

The authors declare that the research was conducted in the absence of any commercial or financial relationships that could be construed as a potential conflict of interest.

## Supplementary Material

Supplementary Figure 1 RNAseq data for winter-dependent genes and violin plots from the snRNAseq dataset showing their cluster specific expression.
