# Supplementary figures and images for: Immunologic Profiling of the Atlantic Salmon Gill by Single Nuclei Transcriptomics
Source: Front Immunol. 2021 May 4;12:669889. doi: 10.3389/fimmu.2021.669889 (PMC8129531; doi:10.3389/fimmu.2021.669889)

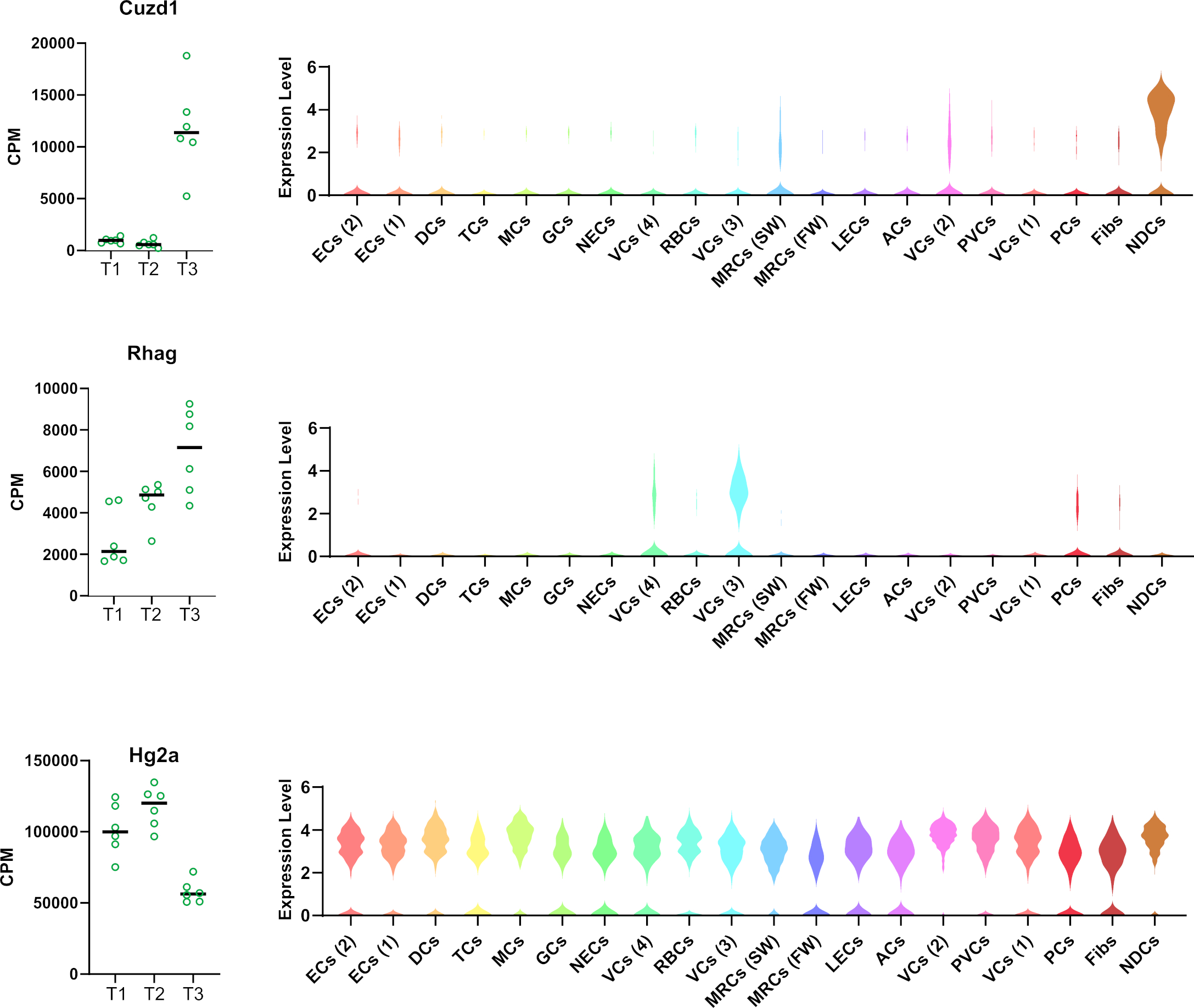

Supplement: Supplementary Figure 1 — RNAseq data for winter-dependent genes and violin plots from the snRNAseq dataset showing their cluster specific expression. [file Image_1.jpeg]
